# Left Atrial Appendage Morphology and Left Atrial Wall Thickness Are Associated with Cardio-Embolic Stroke

**DOI:** 10.3390/jcm9123944

**Published:** 2020-12-05

**Authors:** Agne Adukauskaite, Fabian Barbieri, Thomas Senoner, Fabian Plank, Michael Knoflach, Christian Boehme, Florian Hintringer, Silvana Mueller, Axel Bauer, Gudrun Feuchtner, Wolfgang Dichtl

**Affiliations:** 1 Department of Internal Medicine III, Cardiology and Angiology, Medical University of Innsbruck, 6020 Innsbruck, Austria; fabian.barbieri@i-med.ac.at (F.B.); thomas.senoner@i-med.ac.at (T.S.); fabian.plank@i-med.ac.at (F.P.); florian.hintringer@tirol-kliniken.at (F.H.); silvana.mueller@tirol-kliniken.at (S.M.); axel.bauer@tirol-kliniken.at (A.B.); wolfgang.dichtl@tirol-kliniken.at (W.D.); 2 Department of Neurology, Medical University of Innsbruck, 6020 Innsbruck, Austria; michael.knoflach@i-med.ac.at (M.K.); christian.boehme@i-med.ac.at (C.B.); 3 Department of Radiology Medical University of Innsbruck, 6020 Innsbruck, Austria; gudrun.feuchtner@i-med.ac.at

**Keywords:** left atrial appendage morphology, computed tomography, cardioembolic stroke, atrial fibrillation

## Abstract

Background. New markers for stroke risk stratification in patients with atrial fibrillation (AF) are on demand. Hence, we aimed to investigate the association of left atrial appendage (LAA) and left atrium (LA) morphological parameters in patients with cardio-embolic (CE) stroke due to AF in comparison to controls without stroke. Methods: A retrospective analysis of cardiac computed tomography angiography (CTA) examinations performed between 2006 and 2017 for clinical indications in 158 patients (median age 65 (54–73) years, 48.7% females) was conducted: 56 patients with CE stroke were compared to 102 controls not differing in gender, body mass index (BMI) and CHA_2_DS_2_-VASc score. Results: On multivariable regression analysis adjusted for CHA_2_DS_2_-VASc score and LA diameter CE stroke was independently associated with the following parameters: windsock LAA type (OR 2.55; CI: 1.04–6.26, *p* = 0.041), a greater lobe number (OR 1.54; CI: 1.13–2.10, *p* = 0.006), a greater LAA ostium area (OR 1.88; CI: 1.38–2.55, *p* < 0.001) and a greater left atrium wall thickness (LAWT) in the middle and right part, measured along the anterior LA wall in the axial plane (respectively, OR 1.94; CI: 1.26–3.0, *p* = 0.003 and OR 1.57; CI: 1.07–2.31, *p* = 0.021). Conclusions: The windsock LAA type, a greater LAA lobe number, a larger LAA ostium and a greater LAWT are associated with CE stroke. These CTA parameters could improve risk stratification for thromboembolic stroke.

## 1. Introduction

Stroke is one of the leading causes of functional impairment, morbidity, mortality, and socioeconomic burden worldwide [1]. More than a quarter of strokes are caused by atrial fibrillation (AF) [1], and this subgroup of strokes generally represents the most severe ones [2]. There is growing evidence that atrial cardiopathy (characterized by fibrosis, enlarged size, electrocardiographic changes such as premature atrial contractions, increased plasma levels of natriuretic peptides) as well as a distinctive left atrial appendage morphology (LAA) are associated with stroke even without known AF [3]. 

LAA morphology as well as a greater left atrial wall thickness (LAWT) have already been associated with cryptogenic stroke [4]. The LAA acts as a decompression chamber and as a contractile reservoir during left ventricular systole and high left atrial (LA) pressure [5]. The LAA may also influence LA pressure by producing atrial natriuretic factor [6].

To determine LAA anatomy we used 4 morphological types (windsock, chicken-wing, cauliflower, and cactus) as previously described by Wang et al. [7] and di Biase et al. [8]. 

Due to its distinctive anatomy and hemodynamic situation, the LAA accounts for the vast majority of sources for thrombi in cardio-embolic (CE) strokes caused by non-valvular AF [9,10]. A recent study suggests that in patients with AF undergoing transesophageal echocardiography (TEE) before cardioversion, even 99% of intracardiac thrombi are found in the LAA [11]. However, little is known which LAA parameters are associated with the highest CE stroke risk in AF patients. 

Therefore, the aim of our study was to assess novel LAA and LA morphological and quantitative computed tomography angiography (CTA) parameters, such as LAA ostium angulation, LAA tip angulation (stratified into three layers: ≤90°, 91–110°, >110°) as well as left atrial wall thickness and their association with the history of CE stroke in a retrospective cohort study.

## 2. Experimental Section

### 2.1. Patient Population

Our hospital database was screened for patients with ischemic stroke or transient ischemia attack (TIA) and a clinically indicated cardiac computed tomography (CTA) between 2006 and 2017. Two-hundred-forty patients were identified and enrolled into this retrospective single-center study. Clinical indications for coronary CTA were suspected coronary artery disease or pre-procedural evaluation prior to pulmonary vein isolation. The timespan between CTA and stroke was on average 15.5 months and patients were enrolled if they had both: a CTA and stroke/TIA event independent of the fact, what occurred first, CTA or stroke/TIA event.

Out of those 240 patients 158 patients met the inclusion criteria and were finally enrolled. The inclusion criteria for the CE stroke cohort (*n* = 56) was cardio-embolic stroke or TIA due to AF, defined by the Trial of Org 10,172 in Acute Stroke Treatment (TOAST) criteria [12] and an already available cardiac CTA of good quality. These patients were further compared to controls without stroke. 

The inclusion criteria for the control group (*n* = 102) were no history of stroke or TIA with also available cardiac CTA.

Additional inclusion criteria for both, CE stroke and control group, was a high thromboembolic risk with a CHA_2_DS_2_-VASc score ≥ 2.

Exclusion criteria in both groups were hyperthyroidism and/or advanced renal insufficiency with eGFR < 30 mL/min/1.73 m^2^.

### 2.2. Computed Tomography Angiography (CTA)

Coronary CTA was performed with either a 64-slice computed CTA or a 128-slice dual source CTA (Definition FLASH, Siemens, Forchheim, Germany) with a detector collimation of 2 × 64 × 0.6 mm and a z-flying spot and a rotation time of 0.33 s and 0.28 s, respectively. Prospective ECG-triggering was used in regular heart rates < 65 bpm (70% of RR-interval). At a heart rate of > 65 bpm and at irregular heart rate, retrospective ECG-gating was applied.

An iodine contrast agent (Iopromide, Ultravist 370™, Bayer, IN, USA) was injected intravenously (flow rate 4–6 mL/s + 40 cc saline chaser), triggered into arterial phase (bolus tracking; 100 HU threshold; ascending aorta). Contrast volume ranged between 65–120 cc according to the individual patient characteristics. Axial images were reconstructed with 0.75 mm slice width (increment 0.4/medium-smooth kernel B26f) during best diastolic and systolic phase. The LAA and LA morphology was evaluated using a SyngoVia^TM^ (Siemens, Forchheim, Germany) software.

### 2.3. LAA Morphological Measurements by CTA

**Figure 1:**
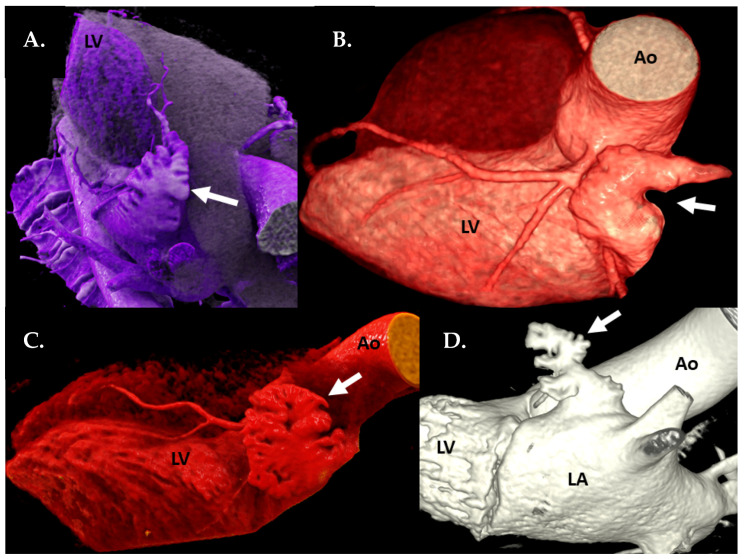
Computed tomography angiography (CTA): 3D volume rendering technique (VRT) reconstructions of the 4 LAA types (white arrows): (**A**) cactus, (**B**) chicken-wing, (**C**) cauliflower, (**D**) windsock. Ao—ascending aorta, LA—left atrium, LV—left ventricle.

Using cardiac CTA, 3D volume rendering (VR) reconstructions were applied to determine the 4 LAA shapes (Figure 1):

1. Windsock type has one long dominant lobe. There might be smaller secondary lobes, extending in inferior direction.
2. Chicken-wing type has a distinctive bend in the proximal or middle part of the main lobe, folding back on the LA or LAA itself. This LAA type might have secondary lobes.
3. Cauliflower lacks a dominant lobe but has a more irregular internal anatomy with multiple lobes, extending primarily to the sides instead of to the length.
4. Cactus has a dominant central lobe with secondary lobes extending to both, inferior and superior directions, in contrast to Windsock type having only inferiorly extending secondary lobes.

#### Novel and Known Parameters in CE Stroke Population

**Figure 2:**
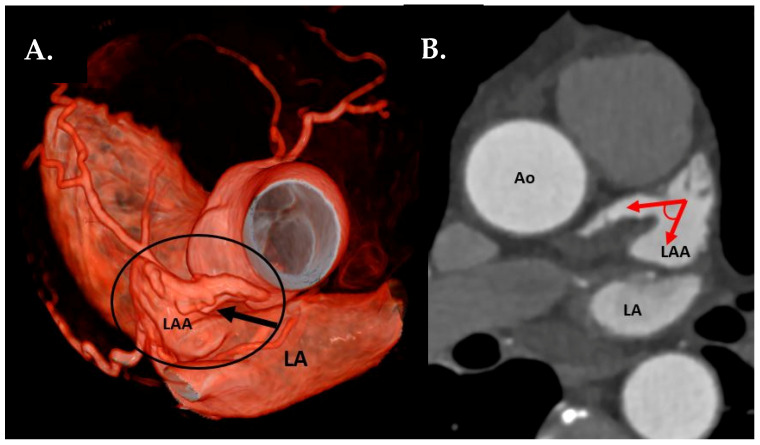
(**A**) CTA 3D-VRT reconstruction of LAA with a sharp tip angulation (≤90°) (black arrow). (**B**) sagittal plane with red arrows delineating sharp LAA tip angulation. Ao—aorta, LA—left atrium, LAA—left atrial appendage.

**Figure 3:**
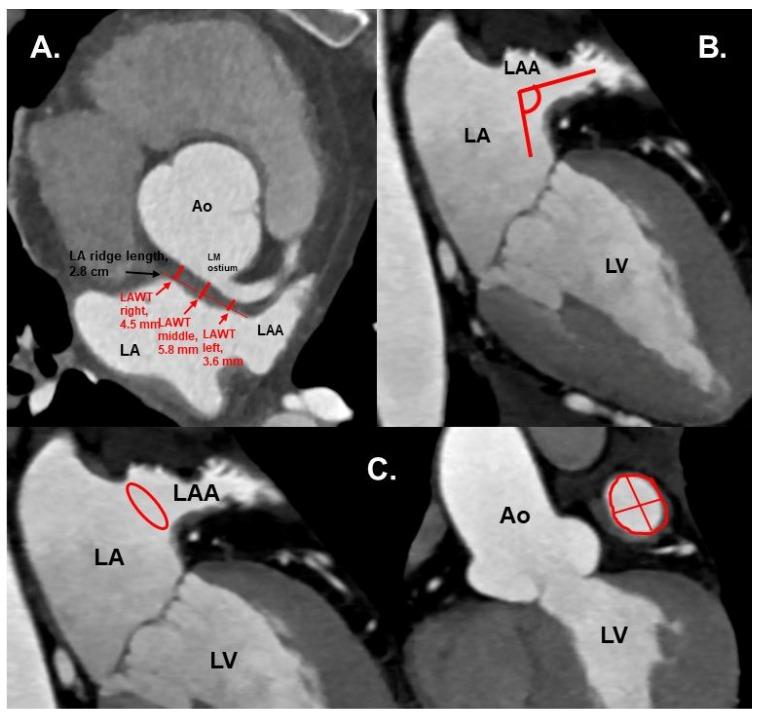
(**A**) Cardiac CTA: axial plane. Red arrows show the LAWT measurements at the left, middle and right points, and the LA ridge length. Measurements were taken at the plane of left main coronary ostium. (**B**) CTA: axial oblique plane, showing LAA ostium angulation (red lines) to orthogonal plane. (**C**) CTA with axial oblique planes of the heart. Left: LAA ostium area shown in red circle. Right: LAA ostium area (red circle), length and width (red lines). Ao—aorta, LA—left atrium, LAA—left atrial appendage, LAWT—left atrial wall thickness, LM—left main coronary artery, LV—left ventricle.

1. LAA tip angulation was quantified using multiplanar reformation (MPR) and stratified as follows: ≤90° tip (Figure 2), 91–110°, >110°.
2. Left atrial anterior wall thickness (LAWT), also called LA ridge, was measured at three points antero-posteriorly (left—being the closest point to the LAA ostium, middle and right—the point of the LA anterior wall, which is most distant to the LAA) and the mean of the three points was calculated; LA ridge length was defined as the medio-lateral length of the LA ridge. These parameters were measured in axial plane at the left main (LM) coronary artery ostium and LAA ostium level using a digital caliper (Figure 3a).
3. Number of LAA lobes using 3D—VR according to Abbara et al. [13]
4. LAA ostium parameters using interactive oblique MPR:
  - ostium angulation (to a sagittal plane) (Figure 3b),
  - ostium dimensions: length (the longest diameter of LAA in mm), width (the shortest diameter of LAA, perpendicular to the length, in mm) and area in cm^2^ (measured on a corresponding orthogonal view) (Figure 3c).
5. LAA filling defects as thrombus.
6. LA volume dimension (diameter measured between aortic root and posterior wall of the LA, in cm).

The quantitative measurements were conducted by 1 observer and the final interpretation was supervised by an experienced observer with >10 years of cardiac computer tomography (CT) experience (Society of Cardiovascular Computed Tomography level III). 

### 2.4. Ethics

The retrospective study protocol was approved by the local ethical committee of the Medical University of Innsbruck (registered number: 1082/2019). 

### 2.5. Statistical Analysis 

Categorical variables are given as absolute numbers and percentages. Continuous variables are provided as median and interquartile range (IQR). Group-specific differences were calculated by using Chi-square tests for categorical variables and either independent samples t-test or the Mann—Whitney U test for continuous variables based on their distribution. The distribution was evaluated by the Shapiro–Wilk test. A multivariable analysis was performed for adjusting of possible confounding. Binary logistic regression models were designed using predefined parameters. A diagnostic and prognostic accuracy of certain LAA parameters was assessed with receiver operating statistics curve (ROC) analysis with the area under the curve (AUC) and given with 95% confidence intervals (CI). The appropriate cut off values of the indexes were determined by the maximum of Youden’s index (sensitivity + specificity − 1, with the highest sensitivity and specificity combination). Statistical analysis was conducted using IBM SPSS, version 24 (IBM Corporation, Armonk, NY, USA). *P*-values ≤ 0.05 were considered statistically significant.

## 3. Results

Clinical patient characteristics are presented in Table 1. CHA_2_DS_2_-VASc score did not differ significantly between the two groups (*p* = 0.078), though patients in the CE stroke group were significantly older and had a higher prevalence of arterial hypertension and hyperlipidemia.

In the CE group 8 (15%) patients were already on oral anticoagulation (mostly on vitamin-K antagonists) at the time-point of stroke diagnosis.

Table 2 shows LAA and LA morphology by CTA in patients with CE stroke compared to controls. The prevalence of cactus, cauliflower, chicken-wing and windsock type was with 10.7%, 21.4%, 37.5%, and 30.4% for the CE stroke group (*n* = 56), slightly divergent from the non-stroke group with 16.7%, 29.4%, 41.2%, 12.7%, respectively (*p* = 0.051). The windsock type LAA was associated with CE stroke in the univariable analysis (OR 2.98; CI: 1.32–6.74, *p* = 0.009) and remained associated in the multivariable regression analysis after adjusting for CHA_2_DS_2_-VASc score and the LA diameter (OR 2.55; CI: 1.04–6.26, *p* = 0.041). 

A greater number of lobes as well as a larger LAA ostium area were associated with CE stroke in univariable analysis (OR 1.4; CI: 1.1–1.84, *p* = 0.014 and OR 1.88; CI: 1.38–2.55, *p* < 0.001, respectively) and in a multivariable analysis after adjustment for CHA_2_DS_2_-VASc score and LA diameter (OR 1.54; CI: 1.13–2.10, *p* = 0.006 and OR 2.16; CI: 1.61–2.88, *p* < 0.001, respectively). In ROC analysis, LAA ostium area with a cut-off ≥ 3.96 cm^2^ proved to be associated with CE stroke (AUC 0.77, CI: 0.69–0.85, *p* < 0.001, sensitivity 67.3%, specificity 80.4%).

Both LAA ostium diameters (length and width) were associated with CE stroke in a univariable analysis (OR 1.22; CI: 1.12–1.32, *p* < 0.001 and OR 1.2; CI: 1.11–1.3, *p* < 0.001, respectively) and in a multivariable analysis after adjusting for CHA_2_DS_2_-VASc score and LA diameter (OR 1.17; CI: 1.07–1.28, *p* < 0.001 and OR 1.13; CI: 1.03–1.23, *p* = 0.009, respectively).

LAWT in the middle, right parts and the mean of all three parts (left, middle, and right) were associated with CE stroke on univariable analysis (OR 1.8; CI: 1.24–2.62, *p* = 0.002, OR 1.55; CI: 1.08–2.21, *p* = 0.017 and OR 1.68; CI: 1.05–2.71, *p* = 0.032, respectively), as well as after adjusting for CHA_2_DS_2_-VASc score and LA diameter in the multivariable regression model (OR 1.94; CI: 1.26–3.0, *p* = 0.003 and OR 1.57; CI: 1.07–2.31, *p* = 0.021, respectively), whereas the mean LAWT only showed a borderline significant association (OR 1.66; CI: 1.0–2.75, *p* = 0.052). 

14 out of 56 (25%) CE patients had LAA filling defects in CTA. Out of those 13 were confirmed as thrombus by TEE.

The anteroposterior LA diameter was larger in the CE group (*p* < 0.001).

## 4. Discussion

Our case-controlled retrospective cohort study reveals novel LAA parameters associated with CE stroke.

For the first time, we show that only the windsock LAA type is independently associated with CE stroke in the presence of AF. A straight-shaped LAA lumen seems to provide favorable conditions for a thrombus to detach and pass through the LAA into the systemic circulation. This finding is new and in accordance with the study results of di Biase et al. [8], who showed, that a non-chicken wing LAA type was associated with CE stroke (*n* = 78) with the windsock type among others having the highest CE stroke prevalence. Moreover, according to his study, patients with a windsock type were 4.5 times more likely to have had a stroke/TIA (*p* = 0.038) in comparison to patients with chicken-wing [8]. This has been confirmed by a meta-analysis of Lupercio et al., showing that AF patients with a chicken-wing LAA type have lower risk to suffer from thromboembolism as compared to AF patients with a non-chicken-wing type [14]. In contrast, a study of Kimura et al. showed that the cauliflower morphology predicts CE stroke (*n* = 30, OR 3.355, *p* = 0.017) in low risk patients (CHA_2_DS_2_-VASc score 0–1) [15]. This might be explained that cauliflower LAAs have a greater number of secondary lobes with trabeculations [15] providing an advantageous architecture for thrombus formation. Our data are in line with this study, since we found an increasing LAA lobe number independently associated with CE stroke. This study finding in line with previously published data using older and non-gated CT technology, which showed that more extensive LAA trabeculations (possibly indicating more lobes on TEE and non-gated CT scans and older CT technology) are associated with LAA thrombus development [16] as well as CE stroke in AF patients [17]. We used high-end CT technology (128 dual source) and ECG-gating at a high spatial resolution in submillimeter range, allowing for improved image quality and better visualization improving better discrimination of trabeculations.

An enlarged LAA ostium may relate to an enlarged LA or LAA itself and hence result in impaired LAA emptying function. These conditions—all together—could facilitate blood stasis and thrombus formation.

Further, a larger LAA ostium area, length and width were found to be associated with CE stroke, most likely attenuating to initially described mechanism on facilitated thrombus-evacuation through a larger LAA ostium.

A greater LAWT in the middle and right parts was independently associated with CE stroke. The mean of the three points (left, middle, and right) showed a borderline association, which supports the concept of positive left atrial wall remodeling in AF, since a greater LAWT indirectly signals remodeling of LA [18]. Since LAWT in principal has a high variation along the entire wall, our data identified two specific measurement sites (middle and right part) as the best points highest predictive for CE stroke, whereas the mean was not as powerfully associated with stroke.

Our study is the first to show an association between LAWT and CE stroke (independent of LA diameter and CHA_2_DS_2_-VASc score). Our previous study, similarly, demonstrated a greater LAWT to be independently associated with cryptogenic stroke, which was presumably caused by occult AF in at least a third of all cases [4]. This parameter appears to be in line with the known biomarkers, which are associated with atrial cardiopathy and stroke, such as LAA shape, size, and LA enlargement [3].

### Limitations

- We acknowledge the inherent limitations related to the retrospective study design. However, we have adjusted our analysis for the major risk factors to avoid bias as rigorously as possible.
- The two groups differed slightly in age and prevalence of arterial hypertension, hyperlipidemia and atrial fibrillation. However, the thromboembolic risk did not differ according to the CHA_2_DS_2_-VASc score, which covers all main stroke risk factors (using a cut-off of ≥2). In addition, the CTA measurements were adjusted for CHA_2_DS_2_-VASc score in the multivariable regression analysis in order to compensate for the minor bias introduced by the risk factors listed above.
- Another possible control group could have been AF patients without a history of stroke. However, several studies comparing LAA morphology in AF patients with stroke versus without stroke are already available. Hence, our study design brings new aspects to current literature.

## 5. Conclusions

Our study data show that windsock LAA type, a greater lobe number, a larger LAA ostium and a new parameter—greater LAWT—were independently associated with CE stroke in AF patients with high thromboembolic risk. Hence, they add incremental value for stroke risk stratification, independent of the CHA2DS2-VASc score.

In patients who had undergone CTA for clinical indications (e.g., coronary artery disease evaluation or planning of left atrial ablation) these LAA parameters may provide additional information regarding thromboembolic risk stratification and consecutive decisions regarding the anticoagulation scheme.

## Figures and Tables

**Table 1:** Clinical patients’ characteristics in cardio-embolic (CE) stroke group vs. controls.

Characteristic	CE Stroke (*n* = 56)	Non-Stroke (*n* = 102)	*p* Value
Females	24 (42.9%)	53 (52.0%)	0.273
Age, y	71.5 (65–76.6)	57.5 (50–70)	<0.001
BMI, kg/m^2^	26.1 (22.8–28.6)	26 (23.3–30.1)	0.360
CHA_2_DS_2_-VASc score	3 (1–4)	2 (1–3)	0.078
Atrial fibrillation	56 (100%)	4 (3.9%)	<0.001
Arterial hypertension	53 (96.4%)	54 (56.3%)	<0.001
Diabetes mellitus, type 2	4 (7.3%)	11 (11.5%)	0.574
Hyperlipidemia	39 (70.9%)	42 (43.8%)	0.001
Obesity	10 (17.9%)	25 (27.5%)	0.233

Results are given in median ± (interquartile range (IQR) or absolute numbers and percentages. BMI—body mass index, y—years.

**Table 2:** LAA and LA morphology in patients with cardio-embolic (CE) stroke compared to patients without stroke.

Parameter	CE Stroke (*n* = 56)	Non-Stroke (*n* = 102)	*p* Value
Cactus (%)	6 (10.7)	17 (16.7)	0.179
Cauliflower (%)	12 (21.4)	30 (29.4)	0.402
Chicken-wing (%)	21 (37.5)	42 (41.2)	0.652
Windsock (%)	17 (30.4)	13 (12.7)	0.007
Lobe number	3 (2–4)	2 (2–3)	0.020
LAA ostium:			
-area (cm^2^)	4.7 (3.4–6.5)	2.9 (2.5–3.8)	<0.001
-length (mm)	27.0 (23.1–32.8)	22.85 (19.7–24.9)	<0.001
-width (mm)	19.6 (16.2–23.8)	16.05 (13.5–18.6)	<0.001
-angulation (°)	119 (99.0–135.0)	110.5 (95.8–128.0)	0.174
LAA tip ≤ 90° (Harlequin) (%)	19 (33.9)	40 (39.2)	0.511
LAA tip angulation > 110°	20 (36.4)	44 (43.1)	0.304
LAA tip angulation (°)	95 (81.0–134.0)	100.5 (83.3–132.3)	0.972
LAWT in the left part (mm)	2.8 (2.1–3.8)	3.1 (2.5–3.6)	0.208
LAWT in the middle part (mm)	2.7 (2.0–3.2)	2 (1.5–2.5)	<0.001
LAWT in the right part (mm)	2.0 (1.5–2.7)	1.8 (1.3–2.2)	0.037
LAWT mean (mm)	2.6 (2.2–3.0)	2.3 (1.9–2.7)	0.018
length of the LA ridge (cm)	3.0 (2.8–3.4)	3.0 (2.7–3.3)	0.178
LAA filling defect (%)	14 (25)	1 (1)	<0.001
LA width (cm)	4.0 (3.3–4.7)	3.4 (3.1–3.9)	<0.001

Results are given in median ± interquartile range (IQR) or absolute numbers and percentages. LA—left atrium, LAA—left atrial appendage, LAWT—left atrial wall thickness.
